# Performance of four computer-coded verbal autopsy methods for cause of death assignment compared with physician coding on 24,000 deaths in low- and middle-income countries

**DOI:** 10.1186/1741-7015-12-20

**Published:** 2014-02-04

**Authors:** Nikita Desai, Lukasz Aleksandrowicz, Pierre Miasnikof, Ying Lu, Jordana Leitao, Peter Byass, Stephen Tollman, Paul Mee, Dewan Alam, Suresh Kumar Rathi, Abhishek Singh, Rajesh Kumar, Faujdar Ram, Prabhat Jha

**Affiliations:** 1 Centre for Global Heath Research, St. Michael’s Hospital, Dalla Lana School of Public Health, University of Toronto, Toronto Ontario, Canada; 2 Center for the Promotion of Research Involving Innovative Statistical Methodology, Steinhardt School of Culture, Education and Human Development, New York University, New York NY, USA; 3 WHO Collaborating Centre for Verbal Autopsy, Umeå Centre for Global Health Research, Umeå University, Umeå, Sweden; 4 Umeå Centre for Global Health Research, Division of Epidemiology and Global Health, Department of Public Health and Clinical Medicine, Umeå University, Umeå, Sweden; 5 Medical Research Council/Wits University Rural Public Health and Health Transitions Research Unit (Agincourt), School of Public Health, Faculty of Health Sciences, University of the Witwatersrand, Johannesburg, South Africa; 6 International Network for the Demographic Evaluation of Populations and Their Health (INDEPTH) Network, Accra, Ghana; 7 International Centre for Diarrhoeal Disease Research, Bangladesh (ICDDR,B), Dhaka, Bangladesh; 8 International Institute for Population Sciences, Mumbai, Maharashtra, India; 9 School of Public Health, Post Graduate Institute of Medical Research and Education, Chandigarh, India

**Keywords:** Causes of death, Computer-coded verbal autopsy (CCVA), InterVA-4, King-Lu, Physician-certified verbal autopsy (PCVA), Random forest, Tariff method, Validation, Verbal autopsy

## Abstract

**Background:**

Physician-coded verbal autopsy (PCVA) is the most widely used method to determine causes of death (CODs) in countries where medical certification of death is uncommon. Computer-coded verbal autopsy (CCVA) methods have been proposed as a faster and cheaper alternative to PCVA, though they have not been widely compared to PCVA or to each other.

**Methods:**

We compared the performance of open-source random forest, open-source tariff method, InterVA-4, and the King-Lu method to PCVA on five datasets comprising over 24,000 verbal autopsies from low- and middle-income countries. Metrics to assess performance were positive predictive value and partial chance-corrected concordance at the individual level, and cause-specific mortality fraction accuracy and cause-specific mortality fraction error at the population level.

**Results:**

The positive predictive value for the most probable COD predicted by the four CCVA methods averaged about 43% to 44% across the datasets. The average positive predictive value improved for the top three most probable CODs, with greater improvements for open-source random forest (69%) and open-source tariff method (68%) than for InterVA-4 (62%). The average partial chance-corrected concordance for the most probable COD predicted by the open-source random forest, open-source tariff method and InterVA-4 were 41%, 40% and 41%, respectively, with better results for the top three most probable CODs. Performance generally improved with larger datasets. At the population level, the King-Lu method had the highest average cause-specific mortality fraction accuracy across all five datasets (91%), followed by InterVA-4 (72% across three datasets), open-source random forest (71%) and open-source tariff method (54%).

**Conclusions:**

On an individual level, no single method was able to replicate the physician assignment of COD more than about half the time. At the population level, the King-Lu method was the best method to estimate cause-specific mortality fractions, though it does not assign individual CODs. Future testing should focus on combining different computer-coded verbal autopsy tools, paired with PCVA strengths. This includes using open-source tools applied to larger and varied datasets (especially those including a random sample of deaths drawn from the population), so as to establish the performance for age- and sex-specific CODs.

## Background

Verbal autopsy (VA) is used in areas with limited medical certification of death to obtain information on causes of death (CODs) [1-3]. VA tools typically consist of a structured survey administered to a close relative or associate of the deceased by a trained field worker, to record the signs and symptoms that occurred before death. This information is used to assign the most probable COD, most often via physician-certified verbal autopsy coding (PCVA).

PCVA has limitations in inter- and intra-observer differences in coding [3], but remains widely useful, particularly in establishing population-based estimates of the major CODs [1]. There has been interest in the use of computer-coded VA (CCVA) methods to automate COD assignment [3]. CCVA methods are, in theory, cheaper, faster and more consistent over time - but their performance against PCVA and against each other has not yet been assessed reliably.

Here, we compare the performance of four of the most promising CCVA methods - InterVA-4, King-Lu (KL), open source random forest (ORF) and open source tariff method (OTM) - across VA studies in several countries, covering more than 24,000 deaths, including community- and hospital-based deaths (Table 1). We define performance by their ability to replicate physician coding.

**Table 1:** Dataset specifications

**Variable**	**China**	**Institute for Health Metrics and Evaluation**	**Million Death Study**	**Agincourt**	**Matlab**
Region	China	N/A^a^	India	South Africa	Bangladesh
Sample size	1,502	1,556	12,225	5,823	3,270
Ages	15+ years	15 to 105 years	1 to 59 months	15 to 64 years	20 to 64 years
Number of CODs	31	32	15	17	17
Population	Hospital deaths	Hospital deaths	Community deaths	Community deaths	Community deaths
Proportion ill-defined deaths^b^	0%	0%	3%	12%	2%
Physician coding	Coding by a panel of three physicians assisted with medical records and diagnostic tests	Coding by one physician assisted with medical records and diagnostic tests	Dual, independent coding of VA records, disagreements resolved by reconciliation, and for remaining cases by adjudication by a third physician	Dual, independent coding of VA records, disagreements resolved by third physician.	Single physician re-coding of VA records after initial coding by another physician.

All VA data in the Million Death Study, Agincourt and Matlab studies were collected by non-medical field staff, and coded by medical staff. ^a^The full IHME hospital-based dataset includes 12,000 VA records from India, Philippines, Tanzania and Mexico and was released after this paper went to press; correspondence with the study team suggested these data were from Bangladesh but the full details of the 1,556 deaths are not published. ^b^Ill-defined deaths are International Classification of Diseases-10 codes R95-R99. VA, verbal autopsy.

## Methods

### Datasets

Table 1 summarizes important features of the five VA datasets. The datasets from the Matlab study in Bangladesh [4], from China [5], from Agincourt, South Africa [6], and a published study of the Institute for Health Metrics and Evaluation (IHME) [7,8] comprised adult deaths. The Indian Million Death Study (MDS) [9,10] included only child deaths from ages 1 to 59 months. Each study used different field procedures, although with similar collection of major symptoms for each death. Physician coding guidelines and procedures also varied but generally involved at least one doctor examining each record. The China and IHME datasets involved physician coding of hospital-based deaths with additional information on medical histories and diagnostic tests. The four CCVA methods were tested on all five datasets with each study’s PCVA assignment as the reference standard. We could not test InterVA-4 on the China and IHME data due to the unavailability of a data dictionary at the time of analysis.

### Computer-coded verbal autopsy methods

#### InterVA-4

InterVA-4 assigns CODs using a Bayesian model with *a priori* probabilities based on expert consensus. InterVA-4 version 4.02 was used in this study, and the program with a full description of its logic can be freely obtained online [11].

#### Open-source random forest

**Figure 1:**
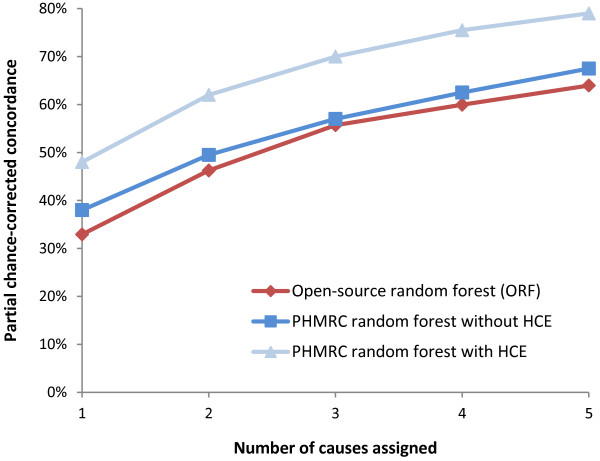
**Comparison of open-source random forest to IHME random forest.** The IHME random forest was tested on a set of IHME hospital-based data, both with and without health care experience (HCE) variables. HCE variables are binary questions on previous medically diagnosed conditions (including high blood pressure, tuberculosis, cancer), and details transcribed from the respondents’ medical records. Our IHME subset contained some, but not all, HCE variables. The ORF performance was similar to the IHME random forest method on the full hospital-based dataset without HCE variables, but performed less well when HCE variables were included [12]. HCE, health care experience; IHME, Institute for Health Metrics and Evaluation; ORF, open-source random forest.

The ORF is a data-driven, probabilistic method that builds upon a similar tool published by IHME [12]. Random forest and tariff methods have been described as having unrivaled performance against all other VA coding methods [12]. However, at the time of writing, these two methods were not publicly available, and their results have not yet been independently replicated. We thus ‘reverse-engineered’ these two methods into open-source tools (details are in Additional file 1). An independent programming expert reviewed the algorithm to assess replication of the IHME method (to the extent of the published details), and we compared the ORF performance on the IHME data available to us to the published results of the IHME methods (Figure 1). The ORF showed very similar trends to those from the IHME random forest, though differences were to be expected due to the unavailability of the full IHME hospital-based dataset [7]. In addition, 96 symptom indicators were used by ORF whereas the IHME method used only the top 40 most-predictive symptoms, the details of which were unavailable [12].

#### Open-source tariff method

**Figure 2:**
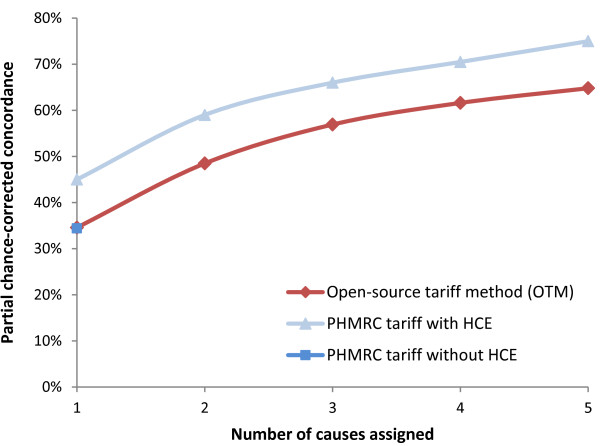
**Comparison of open-source tariff method to IHME tariff method.** The IHME random forest was tested on a set of IHME hospital-based data, both with and without health care experience (HCE) variables. The ORF was tested on a subset of the full IHME data, containing some, but not all, HCE variables. The OTM performed almost exactly as the similar IHME method on the full hospital-based dataset without HCE variables (for the top cause), but less well than the same IHME analysis with HCE variables. Note that results for the full IHME dataset without HCE were only available for the top assigned cause [13]. HCE, health care experience; IHME, Institute for Health Metrics and Evaluation; OTM, open-source tariff method.

The OTM is a data-driven, probabilistic method that builds upon that published by IHME [13]. The OTM performance on the IHME data available to us was comparable to the results of the IHME method (Figure 2). The resulting differences may be due to similar factors as those mentioned in the ORF description above.

#### King-Lu method

The KL method directly estimates cause-specific mortality fractions (CSMFs) without assigning individual CODs. Version 0.9-2.12 was used, for which a full description of the method and discussions of its performance have been published [5], and which is available for download [14].

### Testing

#### Dataset splits and resampling

InterVA-4 uses pre-assigned Bayesian probabilities to assign the COD and thus does not require a training component. The remaining three methods required a training component, consisting of a subset of the original data with assigned CODs, from which the method learned the cause-specific symptom profiles. The trained algorithm was then used to predict CODs in the test dataset.

Table 2 describes the training and testing samples used in the comparisons. Three sample sizes of the datasets were used to highlight changes in performance based on varying dataset sizes (training/testing splits of 1,100/400 and 1,100/1,100 cases, and a split of the full dataset into an equal number of training and testing cases, whose size varied by dataset.) The IHME and China datasets were relatively small (approximately 1,500 cases), which only allowed for the 1100/400 split. Each of the split sizes above were randomly repeated (random splits of the original dataset into the specified number of training and testing cases) 30 times. However, the performance of the methods converged (that is, did not alter by more than 0.5% compared to the average of any previous resamples [15]) well before the full 30 resamples were run.

**Table 2:** Description of testing on multiple computer-coded verbal autopsy methods and datasets

**Dataset**	**Training/testing cases**	**Number of diagnostic indicators**			
		**King-Lu**	**Open-source random forest**	**Open-source tariff method s**	**InterVA-4**
**China**	1100 / 400	48	48	48	N/A
**Institute for Health Metrics and Evaluation**	1100 / 400	96	96	96	N/A
**Million Death Study**	1100 / 400	89	89	89	N/A
	1100 / 1100	89	89	89	N/A
	6100 / 6100^a^	89	89	89	245
**Agincourt**	1100 / 400	104	104	104	245^b^
	1100 / 1100	104	104	104	245
	2900 / 2900	104	104	104	245
**Matlab**	1100 / 400	224	224	224	245
	1100 / 1100	224	224	224	245
	1600 / 1600	224	224	224	245

Only the numbers of test cases are applicable for the InterVA-4 analyses, as this method does not require any training cases. Additionally, InterVA-4 requires the input of 245 diagnostic indicators, however as many of these were not available in the given datasets, the number of useable variables was lower than 245. ^a^The MDS dataset used for InterVA-4 contained 552 cases, in which we extracted additional InterVA-4 indicators from the narratives. ^b^Each CCVA method ran 30 resamples for each training/testing split within each dataset, except InterVA-4, which used the following number of re-samples: 1 for MDS data; 8, 7, 6 for Agincourt data splits of 400, 1100, and 2900 test cases; and 10, 10, 10 for Matlab data splits of 400, 1100, and 1600 test cases, respectively.

The required format of input data varied by assignment method. Two slightly modified versions of each original dataset were created; one version to be used for the data-driven methods (ORF, OTM, KL), and another for InterVA-4, which only uses specific indicators [16]. InterVA-4 testing on the MDS dataset used 552 child deaths, which had additional details extracted from the MDS narratives to match the required InterVA-4 inputs. Resampling was not done on these 552 records due to the small sample size.

### Performance metrics

Positive predictive value (PPV) and partial chance-corrected concordance (PCCC) were used to measure agreement for individual COD assignment. PPV is the proportion of assigned CODs that were the ‘true’ COD (hereafter called reference COD), and is a common metric in hospital-based VA comparison studies [17]. PCCC is a variation on the PPV, meant to account for correct COD assignments made purely by chance [15]. As the CCVA methods could assign each VA record several CODs with varying probabilities, we calculated PPV and PCCC for agreement between the reference COD and the CCVA’s most probable COD, and for the three most probable CODs. These two measures were simply interpreted as whether the reference COD matched the CCVA method’s most probable cause, or matched one of the three most probable causes.

At the population level, accuracy was measured using CSMF absolute accuracy and error. CSMF error is the absolute difference between the estimated and reference CSMFs, summed across all causes. CSMF accuracy, as proposed by Murray et al., is determined by dividing the CSMF error by 2(1-minimum(CSMF^true^))*,* and subtracting this term from one [15]. This is meant to account for the variation in number of CODs across comparisons, and the resulting values are relative to the worst possible performance in a dataset (that is, coding every death incorrectly) [15]. CSMF accuracy and CSMF error are interpreted in opposite directions: good performance yields high CSMF accuracy and low CSMF error. We retained PCCC and CSMF accuracy for comparability to previous IHME publications. Additional file 2 summarizes the equations for the four metrics.

## Results

### Individual-level agreement on cause of death

In comparison to physician-assigned causes, the agreement (as measured by PPV) for all CCVA methods for the most probable COD averaged 43% to 44% for all datasets, with the highest PPV being 58% for ORF, followed by 52% for OTM, both on the MDS data (Table 3). The average PPV improved for the top three most probable CODs, with greater improvements for ORF (69%) and OTM (68%) than for InterVA-4 (62%). Similar results were seen using PCCC (Table 4): the average PCCC for the most probable COD across the datasets, using ORF, OTM and InterVA-4, was 41%, 40% and 41%, respectively. The average PCCC improved for the top three most probable CODs to 67%, 62% and 58%, respectively.

**Table 3:** Positive predictive values of computer-coded verbal autopsy methods versus physician-coded verbal autopsy reference standards

	**Test cases**	**Open-source random forest**		**Open-source tariff method**		**InterVA-4**		**Average for top cause (%)**
**Dataset**		**Top (%)**	**Top 3 (%)**	**Top (%)**	**Top 3 (%)**	**Top (%)**	**Top 3 (%)**	
**China**	400	35	57	36	70	N/A	N/A	*36*
**Institute for Health Metrics and Evaluation**	400	33	55	34	53	N/A	N/A	*34*
**Million Death Study**	6100	58	82	52	76	42^a^	63^a^	*51*
**Agincourt**	2900	45	77	42	69	42	58	*43*
**Matlab**	1600	49	74	52	74	48	64	*50*
**Average**		*44*	*69*	*43*	*68*	*44*	*62*	

Top cause represents accuracy of the CCVA method’s most probable cause matching the cause assigned by PCVA; Top 3 represents whether CCVA’s three most probable causes contain the cause assigned by PCVA. Averages calculated across CCVA methods only use results for the top cause. ^a^The Million Death Study dataset used for InterVA-4 contained a sample of 552 cases, in which we extracted additional InterVA-4 indicators from the narratives.

**Table 4:** Partial chance-corrected concordance of computer-coded verbal autopsy methods versus physician-coded verbal autopsy reference standards

**Dataset**	**Test cases**	**Open-source random forest**		**Open-source tariff method**		**InterVA-4**		**Average for top cause (%)**
		**Top (%)**	**Top 3 (%)**	**Top (%)**	**Top 3 (%)**	**Top (%)**	**Top 3 (%)**	
**China**	400	33	55	32	64	N/A	N/A	*33*
**Institute for Health Metrics and Evaluation**	400	31	54	32	48	N/A	N/A	*32*
**Million Death Study**	6100	55	81	48	70	38^a^	60^a^	*47*
**Agincourt**	2900	42	75	38	62	39	56	*40*
**Matlab**	1600	45	72	48	68	45	59	*46*
**Average**		*41*	*67*	*40*	*62*	*41*	*58*	

Top cause represents accuracy of the CCVA method’s most probable cause matching the cause assigned by PCVA; Top 3 represents whether CCVA’s three most probable causes contain the cause assigned by PCVA. Averages calculated across CCVA methods only use results for the top cause. ^a^The Million Death Study dataset used for InterVA-4 contained a sample of 552 cases, in which we attempted to extract additional InterVA-4 indicators from the narratives.

The values of PPV and PCCC rose with larger training and testing datasets, suggesting that their results were partly dependent on having a sufficient number of training cases for each COD. The confidence intervals for these metrics were narrow as they mostly represented random resampling, and did not express the true underlying uncertainty in the data arising from misclassification of causes. Additional file 3 provides detailed results for each of the four metrics, including the confidence intervals.

### Population-level agreement on cause-specific mortality fraction

KL had the best average CSMF accuracy across all five datasets (91%), followed by InterVA-4 (72% across three datasets), ORF (71%) and OTM (54%). Except for KL, the remaining CCVA methods traded best performance by dataset, with no clear trend (Table 5). CSMF error yielded similar results, with KL having the lowest error scores (Additional file 3).

**Table 5:** Cause-specific mortality fraction accuracy of computer-coded verbal autopsy methods versus physician-coded verbal autopsy reference standards

**Datasets**	**Test cases**	**King-Lu (%)**	**Open-source random forest (%)**	**Open-source tariff method (%)**	**InterVA-4 (%)**	**Average (%)**
**China**	400	84	79	75	N/A	*79*
**Institute for Health Metrics and Evaluation**	400	88	73	63	N/A	*75*
**Million Death Study**	6100	96	64	33	70^a^	*66*
**Agincourt**	2900	94	72	38	75	*70*
**Matlab**	1600	95	69	59	72	*74*
**Average**		*91*	*71*	*54*	*72*	

^a^The Million Death Study dataset used for InterVA-4 contained a sample of 552 cases, in which we attempted to extract additional InterVA-4 indicators from the narratives.

Using the MDS data, KL had the closest similarity to the ranking of population-level CODs as compared to PCVA, with the top three causes in children under 5 years being the same (acute respiratory infection, diarrheal diseases, and other and unspecified infections; Additional file 3). ORF ended up with the same top three, but ranked other and unspecified infections ahead of acute respiratory infections, and ahead of diarrheal diseases. In the Agincourt data, KL performed better than ORF, matching the top three causes but not in the same ranking as PCVA. By contrast, ORF, somewhat inexplicably, ranked maternal deaths as the second most common COD. In the Matlab data, both KL and ORF showed similar performance in ranking CODs, accurately matching the top three causes. Results for InterVA-4 varied across the comparisons.

## Discussion

This is the largest comparison study yet done of CCVA and PCVA methods. We found that, at an individual level, ORF, OTM and InterVA-4 replicated the coding of physicians comparably, but that the average agreement level for the leading COD was about 50%. Agreement with the physician-assigned code rose substantially for all CCVA methods if the three most probable CODs were considered, and generally improved with larger subsets within a given study. On a population level, the KL method performed best in terms of CSMF accuracy and error, and replicated the CSMF distribution of PCVA in the original datasets fairly well. ORF did not outperform KL, even on the IHME dataset, and did not perform better than InterVA-4, despite claims to the contrary [18]. At the individual level, InterVA-4, which does require training on a dataset, produced broadly comparable results to the methods that do require training.

Comparison to physician coding as a reference standard poses several methodological challenges. Importantly, our study focused on CCVA replication of physician codes (and errors), and not whether the underlying COD assigned by the physician was correct. Validation of PCVA is limited by the lack of a true reference standard in countries where verbal autopsy is performed [1-3,9]. Nonetheless, PCVA with good quality control can yield useful results on COD distributions in countries where medical certification remains uncommon [1]. The studies we included in the comparisons had physician coding done with reasonably good levels of quality control [4-7,9] as shown by a low proportion of ill-defined deaths. Physician coding that contains large amounts of random errors would reduce agreement on a COD in the dual-physician coding system, and would tend to increase the number of unspecified CODs in the International Classification of Diseases (ICD-10) [19], such as ‘senility’ (ICD-10 code R54) or ‘cause not determined’ (ICD-10 code R99). This would in turn make it harder for CCVA methods to identify specific causes. Moreover, the size of the errors or biases in CCVA methods depends on the inherent errors and biases of PCVA results. This is particularly relevant for machine learning, as its accuracy requires learning on “true” class labels. High misclassification rates in the training set will also affect performance in the testing set across datasets, as noted recently on the full IHME dataset [20]

The performance of each CCVA method at individual assignment improved when trained and tested on a larger number of cases, most likely due to a greater number of cases from which to learn the distinct relationships between specific symptom profiles and CODs. The differences in the field and coding methods across studies would tend to reduce the observed differences in PCVA and CCVA between the various comparisons (and more likely so at the individual level than at the population level). This might have contributed to the observed comparability of the results for the four CCVA methods. With larger studies and more standardized field and physician coding methods, any real, underlying differences between various CCVA methods may become apparent. Finally, we note that InterVA-4 has a threshold of probability for designating the most probable cause (that is, the most probable cause must also have a probability above 50%, otherwise the death is classified as indeterminate), whereas ORF and OTM select the most probable causes without applying any thresholds.

On an individual level, no single method was able to replicate physician assignment of COD more than about half the time. At the population level, the King-Lu method was the best method to estimate CSMFs, though it does not assign individual CODs. However, good population-level agreement accuracy does not guarantee good individual agreement [21,22]. A key methodological feature is the need to avoid the false gold standard of hospital-based deaths [1,3]. Reliance on hospital or urban-based deaths for training of automated methods may lead to learning of symptom patterns and other features that are not representative of populations without medical attention. Indeed, the CSMFs between home and hospital deaths are dissimilar, as demonstrated in India [23].

Our study is the largest cross-country comparison of current CCVA methods versus PCVA, covering about twice as many deaths as an earlier multi-country study [7], and including a mix of various ages, and community and hospital deaths. Nonetheless, we faced certain limitations. First, we could not compare the original IHME random forest and tariff algorithms, though the original methods were re-created to the best of our abilities from the published descriptions [12,13], yielding broadly similar results (Figures 1 and 2). Second, access during the analyses phase to the full IHME hospital-based dataset of 12,000 records would have allowed more robust comparisons. Similarly, the China dataset was also somewhat limited by the small sample size.

## Conclusions

Different CCVA methods have various strengths and weaknesses depending on the study scenario and study objective. An ideal solution could involve a combination of automated methods to obtain robust individual- and population-level estimates. In the medium term, it appears unwise and certainly premature to recommend that automated systems replace physicians in coding VAs. CCVA methods could be used in parallel with physician coding to increase speed, efficiency and quality of coding. Future work may focus on the performance of a combination of various automated methods, and must extend to larger datasets and explore specifics for important age groups (children, maternal, adult), by gender, and across various settings of home- and hospital-based deaths. Future studies need to also place specific emphasis on testing computer based methods on a random sample of deaths in countries, as this would be much more useful in determining the true underlying CSMF at the population level [24].

## Abbreviations

CCVA: computer-coded verbal autopsy; COD: cause of death; CSMF: cause-specific mortality fraction; HCE: health care experience; ICD-10: International Classification of Diseases-10; IHME: Institute for Health Metrics and Evaluation; KL: King-Lu verbal autopsy method; MDS: Million Death Study; ORF: open-source random forest; OTM: open-source tariff method; PCCC: partial chance-corrected concordance; PCVA: physician-certified verbal autopsy; PPV: positive predictive value; VA: verbal autopsy.

## Competing interests

The authors declare that they have no competing interests.

## Authors’ contributions

ND, LA and PM conducted the analysis. All authors contributed to data interpretation and critical revisions of the paper. All authors read and approved the final manuscript.

## Supplementary Material

Additional file 1 **Details of the open-source random forest and tariff methods.** Explanation of the major logical steps of the open-source random forest and tariff methods. Click here for file

Additional file 2 **Description of comparison metrics.** Formulas and explanation of positive predictive value, partial chance-corrected concordance, CSMF error and CSMF accuracy. Click here for file

Additional file 3 **Full results of CCVA comparisons on several dataset splits.** Results presented by CCVA method, dataset, dataset splits, and top and top-three cause of death predictions. Click here for file
