# Supplementary material for: Performance of four computer-coded verbal autopsy methods for cause of death assignment compared with physician coding on 24,000 deaths in low- and middle-income countries
Source: BMC Med. 2014 Feb 4;12:20. doi: 10.1186/1741-7015-12-20 (PMC3912488; doi:10.1186/1741-7015-12-20)
Supplement: Additional file 1 — Details of the open-source random forest and tariff methods. Explanation of the major logical steps of the open-source random forest and tariff methods. [file 1741-7015-12-20-S1.pdf]

Additional file 1: Details of the open-source random forest and tariff methods

Open-source random forest (ORF)

The ORF builds on the random forest method as published by IHME [12]. The method uses the Brieman random forest technique to assign a cause of death to individual verbal autopsy cases. The algorithm is first trained on a sample of the VA dataset, which creates the associations between specific symptom profiles and assigned causes of death, and then is applied to predict the causes in a test set of the data. The process of learning is as follows: firstly, 100 decision trees are made for each pair of possible causes of death (pairwise coupling). The decision trees are constructed from randomly selected indicators from cases within those two causes. The structure of all these trees is what is termed the random forest, and is the basis for prediction in the test cases. Each test case's symptom profile is run through these decision trees. Within a pair coupling, the cause of death chosen by the most decision trees "wins".

	Cause 2	Cause 3	Cause 4	Cause 5	Cause 6
Cause 1	1v.2	1v.3	1v.4	1v.5	1v.6
Cause 2		2v.3	2v.4	2v.5	2v.6
Cause 3			3v.4	3v.5	3v.6
Cause 4				4v.5	4v.6
Cause 5					5v.6

Additional figure1: Example of pairwise coupling technique [12]

The number of cause-specific wins are included in a matrix, which are then ranked for likelihood using a novel ranking technique as described by the IHME [12]. These rankings provide the cause-specific probabilities for each VA test case.

Although Flaxman et. al. claim that the novel ranking method results in better performance, our testing found that better individual level accuracy is obtained by simply assigning the cause that was voted the most number of times in the random forest. We incorporated this change into our open-source random forest.

Open-source tariff method (OTM)

The OTM also builds upon the tariff method as published by IHME [13]. This method is a technique that assigns a weight ("tariff") to each symptom pattern for a given cause of death. For example, cough would carry a much heavier weight for death due to tuberculosis than for death due to maternal conditions. Once a tariff is obtained for each of the set of causes of death in the test dataset, the deaths in the training set receive a corresponding tariff score for each cause of death, based on its specific symptom pattern. The probable causes of death for an individual case are simply those causes with the highest tariff scores.

Flaxman et al. claim that better results can be obtained through a slightly more complicated method for assigning the most probable causes of death; by aligning a test case's tariff score to the closest of cause-specific scores obtained in a resampled training dataset with uniform cause distribution. However, we found that the simpler method of simply assigning the cause of death with the highest tariff score yielded better performance.

Both methods are freely available at www.cghr.org/
