# Supplementary material for: Performance of four computer-coded verbal autopsy methods for cause of death assignment compared with physician coding on 24,000 deaths in low- and middle-income countries
Source: BMC Med. 2014 Feb 4;12:20. doi: 10.1186/1741-7015-12-20 (PMC3912488; doi:10.1186/1741-7015-12-20)
Supplement: Additional file 2 — Description of comparison metrics. Formulas and explanation of positive predictive value, partial chance-corrected concordance, CSMF error and CSMF accuracy. [file 1741-7015-12-20-S2.pdf]

Additional file 2: Description of comparison metrics

Metric	**Equation**	**Explanation**
Positive Predictive Value	$$\mathrm{PPV} = \frac{\mathrm{TP}}{\mathrm{TP} + \mathrm{FP}}$$	Proportion of positive test results that are true positives (correct diagnoses)
Partial Chance-Corrected Concordance [15]	$$\mathrm{PCCC(k)} = \frac{\mathrm{C} - \frac{\mathrm{k}}{\mathrm{N}}}{1 - \frac{\mathrm{k}}{\mathrm{N}}}$$ *C is the fraction of deaths for which the true cause is in the top k assigned causes	Used for probabilistic methods that assign probabilities of multiple causes of death for each VA case
CSMF Absolute Error [15]	$$\sum_{\mathrm{j}=1}^{\mathrm{k}} \left\| \mathrm{CSMF}_{\mathrm{j}}^{\mathrm{true}} - \mathrm{CSMF}_{\mathrm{j}}^{\mathrm{pred}} \right\|$$	Sum of the amount by which the estimated CSMF deviates from the true CSMF, for each of cause of death
CSMF Accuracy [15]	$$\mathrm{CSMFAccuracy} = 1 - \frac{\sum_{\mathrm{j}=1}^{\mathrm{k}} \left\| \mathrm{CSMF}_{\mathrm{j}}^{\mathrm{true}} - \mathrm{CSMF}_{\mathrm{j}}^{\mathrm{pred}} \right\|}{2(1 - \mathrm{Minimum}(\mathrm{CSMF}_{\mathrm{j}}^{\mathrm{true}}))}$$	Similar to CSMF error, but adjusts for a correct diagnosis made by chance; results are relative to the worst possible performance (where each death is incorrectly diagnosed)
